# Reconfigurable Multi-Channel Gas-Sensor Array for Complex Gas Mixture Identification and Fish Freshness Classification

**DOI:** 10.3390/s25196212

**Published:** 2025-10-07

**Authors:** He Wang, Dechao Wang, Hang Zhu, Tianye Yang

**Affiliations:** 1 National Key Laboratory of Automotive Chassis Integration and Bionics, School of Mechanical and Aerospace Engineering, Jilin University, Changchun 130022, China; wang_he19@mails.jlu.edu.cn (H.W.); wangdc23@mails.jlu.edu.cn (D.W.); 2 Key Laboratory of CNC Equipment Reliability, Ministry of Education, Jilin University, Changchun 130022, China

**Keywords:** gas sensor, mixed gases, sensor array, fish freshness

## Abstract

Oxide semiconductor gas sensors are widely used due to their low cost, rapid response, small footprint, and ease of integration. However, in complex gas mixtures their selectivity is often limited by inherent cross-sensitivity. To address this, we developed a reconfigurable sensor-array system that supports up to 12 chemiresistive sensors with four- or six-electrode configurations, independent thermal control, and programmable gas paths. As a representative case study, we designed a customized array for fish-spoilage biomarkers, intentionally leveraging the cross-sensitivity and broad-spectrum responses of metal-oxide sensors. Following principal component analysis (PCA) preprocessing, we evaluated convolutional neural network (CNN), random forest (RF), and particle swarm optimization–tuned support vector machine (PSO-SVM) classifiers. The RF model achieved 94% classification accuracy. Subsequent channel optimization (correlation analysis and feature-importance assessment) reduced the array from 12 to 8 sensors and improved accuracy to 96%, while simplifying the system. These results demonstrate that deliberately leveraging cross-sensitivity within a carefully selected array yields an information-rich odor fingerprint, providing a practical platform for complex gas-mixture identification and food-freshness assessment.

## 1. Introduction

Gas sensors, as a crucial branch of the sensor field, possess the ability to identify information such as gas types and concentrations in the environment and convert this information into electrical signals, thereby achieving quantitative or semi-quantitative detection and alarm purposes for gases [1]. Based on differences in structure and working principles, gas sensors can be classified into oxide semiconductor gas sensors [2,3,4], surface acoustic wave gas sensors [5,6], infrared gas sensors [7,8], electrochemical gas sensors [9,10], and others. Among these, oxide semiconductor gas sensors are widely used due to their high detection sensitivity, low manufacturing cost, rapid response, and long service life. However, their performance also has limitations. In complex environments with coexisting multiple gases, a single oxide gas sensor exhibits cross-sensitivity characteristics, meaning that when multiple gases are present simultaneously, the sensor’s response can be interfered with, making it difficult to accurately identify gas types and concentrations based solely on the response of a single sensor [11,12].To address this issue, researchers have adopted a strategy of constructing sensor arrays, which consist of multiple gas sensors with different characteristics. By leveraging the cross-sensitivity and broad-spectrum response features of these sensors and comprehensively analyzing their responses to mixed gases, accurate identification and detection of gas types and concentrations in mixtures can be achieved [13,14]. Gas sensor array technology has garnered significant attention for its applications in various fields such as food testing, medical diagnosis, and environmental monitoring [15]. Jiang et al. utilized nanoscale soft lithography to fabricate a gas sensor array composed of polymer nanowires. By employing Principal Component Analysis (PCA) to extract and analyze sensor responses, the array demonstrated the capability to monitor various Volatile Organic Compounds (VOCs), including alcohols, ketones, alkanes, and nitrogen-based compounds [16]. Wu et al. constructed a sensor array by doping graphene oxide (GO) with different types of metal ions (e.g., Cu^2+^, Fe^2+^, Ce^3+^, Co^2+^) at varying ratios. The array consisted of eight sensing elements, each exhibiting cross-reactive responses to endogenous VOCs associated with lung cancer. When tested on lung cancer patients and healthy controls in a study of 106 cases, the array—assisted by an artificial neural network algorithm—achieved a diagnostic sensitivity of 95.8% and specificity of 96.0% [17]. Shooshtari et al. created such an array by depositing four different electrodes on a CNT-TiO_2_ film, generating distinct response signatures from a single sensing material due to varied electrode interfacial properties. When integrated with dynamic temperature modulation, this strategy significantly enhanced the discrimination of volatile organic compounds, underscoring that the strategic design of the electrode array is a critical factor, alongside material composition, in determining the overall selectivity and performance of an electronic nose system [18]. Lee et al. developed and fabricated a gas sensor array composed of four distinct CuO-based sensing materials. This array enabled effective detection of acetone in breath, holding potential for early diabetes diagnosis. Using PCA, the array successfully distinguished acetone from other VOCs such as ethanol and formaldehyde, demonstrating excellent selectivity [19]. Lidia et al. designed a sensor array system capable of differentiating artificial aromas of strawberry, grape, and apple. The sensors were prepared via in situ polymerization of polyaniline doped with different acids (hydrochloric acid, camphorsulfonic acid, and dodecylbenzenesulfonic acid). Experimental results showed that the system could detect and discriminate varying concentrations of apple, strawberry, and grape aromas at room temperature and high relative humidity (>55%) [20]. When a sensor array system operates in complex and dynamic gas environments, insufficient diversity in sensor types or mismatched performance parameters among sensors may compromise detection accuracy. Moreover, an unscientific or irrational array design can further degrade performance. Therefore, developing cost-effective, highly accurate, and practical gas sensor array systems—along with tailored sensors for key characteristic gases in specific fields—holds significant practical importance for advancing the industrial application of gas sensor technology.

Fish, known for their high protein and low-fat characteristics, have become an indispensable part of people’s daily diets, with their nutritional value well-documented. However, fish are highly prone to spoilage during post-capture transportation, storage, sales, and processing, which not only significantly affects the taste and quality of fish and their processed products but may also pose potential risks to consumer health. Therefore, accurately determining fish freshness, as a key indicator of fish quality, holds particular significance. In the field of fish freshness detection, traditional methods include sensory evaluation, physical property analysis [21,22,23], chemical analysis [24,25,26], and microbiological testing [27]. In recent years, with the continuous advancement of detection technologies, gas sensor detection technology has emerged as a novel method, demonstrating broad application prospects in the field of food safety. Particularly in fish freshness detection, this technology has gradually become a research hotspot due to its advantages of speed, high sensitivity, and non-destructive testing, garnering widespread attention from both academia and industry. Relevant studies indicate that detecting characteristic volatile gas components produced during storage can effectively assess fish freshness, providing new technical support for food safety inspection [28]. A comparison of fish freshness detection technologies is provided in the Supplementary Materials.

This paper develops a semiconductor gas sensor array system for detecting complex gas mixtures, with the array capable of accommodating up to 12 sensors. To verify the practicality and effectiveness of the array system, fish freshness detection was selected as the application scenario. Three self-developed gas sensors were combined with nine commercial sensors to construct a 12-sensor array system specifically designed for fish freshness detection. On this basis, multiple pattern recognition algorithms were employed to achieve rapid and accurate detection of fish freshness. Additionally, the sensor array was optimized in this study, further enhancing the rationality and detection accuracy of the array system. The results demonstrate that leveraging sensor cross-sensitivity to construct such an array provides a feasible and efficient method for complex gas mixture analysis.

## 2. Design of the Gas Sensor Array System

**Figure 1:**
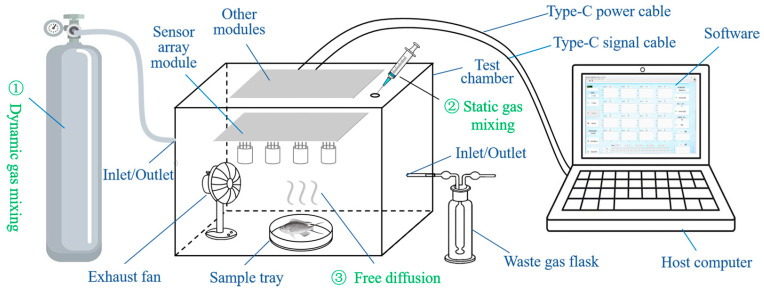
Schematic diagram of the gas sensor array system composition structure.

Figure 1 shows a schematic diagram of the overall structure and composition of the sensor array system. The gas sensor array system consists of three main parts: the hardware module, the software module, and the testing chamber.

**Figure 2:**
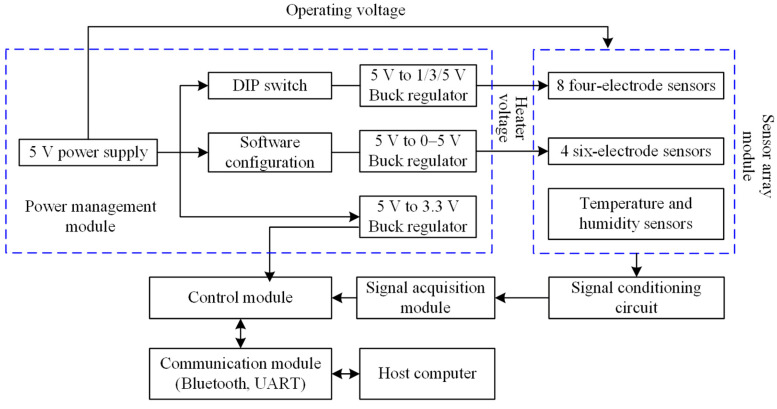
Gas sensor array system hardware architecture.

Figure 2 shows the hardware architecture of the sensor array system. The hardware module primarily includes the control module, communication module, gas sensor detection module, signal acquisition module, and power management module. The gas sensor detection module is distributed on one PCB and placed inside the testing chamber, while the remaining modules are arranged on another PCB located externally to prevent potential corrosion of electronic components caused by reactive gases in the testing environment.

The system uses the STM32F103RCT6 (manufactured by STMicroelectronics, Geneva, Switzerland) as the core controller, which manages multi-sensor data acquisition, processing, display, and storage functions. It also performs analog-to-digital conversion for multiple gas sensors via its built-in ADC module. The communication module includes USB-to-serial converter (CH340E) and Bluetooth module (ECB02S2), providing both wired and wireless communication capabilities.

The gas sensor array module is a critical part of the system. Based on the practical application where gas sensors typically feature four or six electrodes, the designed array can integrate up to eight four-electrode and four six-electrode metal oxide semiconductor gas sensors. This distinction is reflected in the design of the IC sockets for the sensor array module. The hardware architecture offers the potential for integrating other types of sensors (e.g., electrochemical sensors) in the future, where only a redesign of the front-end electronics would be needed to maintain compatibility with the same data processing pipeline. In addition to structural differences, the inherent resistances of various metal oxide semiconductor gas sensors also vary. When the semiconductor materials interact with target gases, physico-chemical reactions occur on the surface, leading to changes in resistance. These changes differ significantly across materials due to their distinct gas-sensitive properties. To convert the resistance variation into a voltage output, a voltage divider circuit with series load resistors is employed. To ensure a stable detection environment, a BME680 sensor is integrated to monitor ambient temperature and humidity.

Since the output signals from gas sensors are generally weak, the acquired signals require amplification, filtering, and level conversion. This system adopts multi-stage amplification and filtering, along with independent ADC sampling, to improve data acquisition accuracy. Analog signals from the gas sensors are fed into the microcontroller via ADC pins, where they are digitized for further processing and analysis. Decoupling capacitors and precision resistors are used to filter out external noise and enhance signal integrity.

The power management module is another core component of the system, responsible for supplying reliable and stable power to all parts. The control, communication, and Bluetooth modules operate at 3.3 V, while the gas sensing elements require 5 V. The heating elements of the gas sensors, which maintain the optimal operating temperature, typically operate within a 0–5 V range. Therefore, the system uses the USB Type-C power control chip along with filtering circuits to provide 5 V power, the AMS1117 voltage regulator to deliver a stable 3.3 V supply, and the DC-DC buck conversion circuit to generate adjustable 0–5 V heating voltage, ensuring the gas sensors remain in their optimal working condition.

A dedicated software system has been developed for the gas sensor array system. It ensures real-time and stable communication between the sensor array hardware and the host computer. The software efficiently processes and displays the detected electrical signals in real time, while also supporting multiple data export formats, greatly facilitating subsequent data processing and analysis. Furthermore, users can flexibly configure key parameters, such as sampling time and sensor heating voltage, through the software interface to accommodate diverse requirements in practical detection scenarios.

Three primary gas distribution methods have been designed for the test chamber. The first is the dynamic distribution method, which incorporates gas inlet and outlet ports of different heights on both sides of the chamber. These ports can be connected to a gas source, with the assignment of the inlet and outlet determined based on the relative density of the target gas compared to air. The second method is static gas distribution, widely used in laboratories due to its convenience. A gas injection port is integrated at the top of the chamber, allowing target gas to be introduced via a syringe. For liquid samples, a micro-syringe may be used to inject the liquid, which subsequently evaporates naturally to fill the chamber. After injection, the port is sealed with a silicone plug to prevent gas leakage. The third approach is free diffusion, wherein the sample to be tested is placed on a sample tray, enabling the gas emitted by the sample to diffuse freely and fill the chamber.

For gas exhaust, two methods are provided. The first, as mentioned above, utilizes the outlet port in the dynamic distribution system. The outlet can be connected to an adsorbent or absorption liquid for gas purification. The second method involves simply releasing the top fasteners and opening the lid for natural ventilation. This approach is relatively convenient and ensures effective air exchange. To enhance exhaust efficiency, the internal fan can be activated during the process. It is recommended that all exhaust operations be conducted in a fume hood or well-ventilated environment.

## 3. Experimental

### 3.1. Materials

A total of 48 fresh common carp, each measuring between 10 and 12 cm in length, were purchased from Taian Zhendong Aquatic Products Co., Ltd. The fish were divided into 24 groups, with two fish per group. Each pair was placed in a separate storage container and assigned a unique identification number for tracking and management purposes. Within each group, one fish was used for gas-sensing detection, while the other was reserved for measuring total volatile basic nitrogen (TVB-N) content, which served as a reference for calibrating the degree of spoilage based on the gas-sensing results. All samples were stored in a refrigerator at a temperature between 0 and 10 °C. For each experiment, only the sample with the designated identification number was taken out for testing, while the remaining samples were kept under refrigeration to maintain their condition until subsequent tests.

### 3.2. Methods

#### 3.2.1. Gas Sensor Array and Its Properties

The selection of a gas sensor array is a complex and meticulous process, involving multiple considerations such as the types of target gases, gas concentration ranges, sensor performance, and the number of sensor electrodes. The sensor selection criteria can be found in the Supplementary Materials. After analyzing and comparing various gas sensor models currently available on the market, a total of 12 gas sensors, as listed in Table 1, were ultimately selected to form a sensor array system for detecting fish freshness and to conduct experimental research. The sensor array system consists of nine commercial gas sensors and three self-developed sensors [29,30,31].

It should be noted that this panel comprises commodity MOS sensors with ppm-level detection ranges. As such, the analytical strategy is designed for freshness-level classification (fresh/sub-fresh/spoiled) calibrated against TVB-N-based labels, relying on multivariate pattern recognition from the sensor array’s response. This approach does not target the trace-level quantification of individual analytes, which represents a deliberate trade-off for achieving a practical, low-cost system.

#### 3.2.2. Gas Sensing Measurement

**Figure 3:**
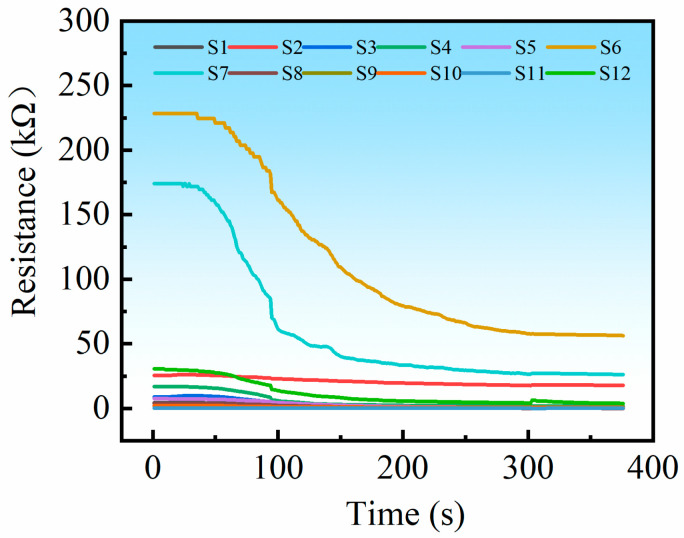
Response curve of the sensor array.

During storage, the samples undergo a gradual decline in freshness, accompanied by microbial growth and the production of various organic gases. As storage time increases, microbial proliferation intensifies, leading to higher concentrations of volatile gases [32]. The samples were tested every 24 h over a period of seven consecutive days. To ensure stable operation of the gas sensors, they were preheated for seven days prior to initial use, in accordance with the commercial sensor specifications. Before the first test each subsequent day, the sensors were preheated for 0.5 h. For each sample, the baseline resistance of the sensor array was first recorded in air for 60 s to calculate the average value. The sample was then quickly removed from the refrigerator, placed into the test chamber, and tightly sealed. Figure 3 shows the response curve of the sensor array to the gases produced by sample spoilage. As volatile compounds from the decaying sample were released, the resistance of the sensor array decreased continuously. After approximately 300 s, the sensor resistance stabilized, and the resistance of the sensor array was recorded in the test gas for 60 s to calculate the average value. The test chamber was then opened, and the sample was returned to the refrigerator for continued storage. The fan inside the test chamber was activated to rapidly purge residual gases. The sensor array system returned to its initial state after desorption and proceeded to the next sample test.

Calculate the response value of the sensor array to the gases produced by sample spoilage as the characteristic indicator for assessing the degree of fish spoilage. The calculation formula is as follows:

(1) $$S=\frac{\bar{R_{a}}}{\bar{R_{g}}}$$

In the formula, *R_a_* denotes the average resistance of the gas sensor in a stable state in air. *R_ᵍ_* represents the average resistance of the gas sensor in a stable state in the test gas.

#### 3.2.3. TVB-N Measurement

TVB-N is a key indicator for evaluating the freshness of aquatic animal products such as fish [33,34]. It refers to the alkaline nitrogenous compounds produced during the decomposition of proteins as a result of enzymatic and bacterial action during spoilage. These substances are volatile, and their concentration increases with the degree of spoilage. TVB-N value is internationally recognized as a core metric for quantitatively assessing the extent of fish spoilage [35,36]. Therefore, the TVB-N content was measured daily for each sample group to accurately calibrate the detection results from the sensor array system.

The semi-micro Kjeldahl method is internationally recognized as the benchmark for determining TVB-N, offering high accuracy and sensitivity. However, it typically requires complex sample pretreatment, skilled operation, and lengthy processing, making it unsuitable for rapid on-site testing. In this study, we developed a multi-gas sensor array to classify fish into three freshness states—fresh, sub-fresh, and spoiled. Because the application scenario does not require extremely high precision, we used the results of a rapid TVB-N detection kit compliant with national standards as the reference, enabling semi-quantitative calibration of the sensor-array outputs.

The TVB-N kit used here was manufactured by Wuxi Yuxiang Biotechnology Co., Ltd. (Wuxi, China), with a detection limit of 15 mg/kg. An appropriate amount of sample was homogenized, and 1g was weighed into a sample cup using a balance. Then, 20 mL of deionized water was added, and the mixture was shaken thoroughly and allowed to stand for 10 min. Subsequently, 1ml of the supernatant was extracted and transferred into a test tube. Two drops of the detection reagent were added to the tube, and the mixture was shaken again. The color development was observed and compared with a standard color card to identify the matching color grade, thereby determining the TVB-N content.

**Figure 4:**
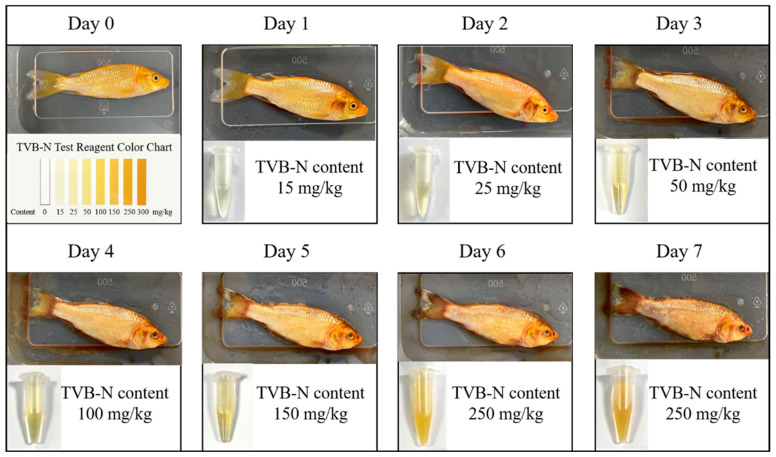
Changes in appearance and TVB-N content during sample spoilage.

Figure 4 illustrates the visual changes and the corresponding TVB-N values of one sample group during spoilage. As shown in the figure, as spoilage progressed, the surface color of the fish gradually turned dull and lost its original bright appearance. Starting from day 3, a small amount of mucus became visible on the surface, which turned cloudy and dirty. The eyes began to sink, indicating a decline in freshness. The TVB-N content at this stage was measured at 50 mg/kg. By day 5, parts of the fish surface had turned dark brown or black, indicating advanced spoilage. The TVB-N content reached 250 mg/kg at this point. According to international standards, the TVB-N content for freshwater fish should not exceed 200 mg/kg, meaning the sample could be identified as spoiled at this stage.

Based on the observed visual changes and TVB-N measurements during the spoilage process, the freshness of the fish was classified into three grades according to TVB-N content, as shown in Table 2: fresh, Sub-fresh, and spoiled.

## 4. Results and Discussion

### 4.1. Principal Component Analysis (PCA)

PCA is a widely used dimensionality reduction technique. It can provide a preliminary assessment of inter-class similarity by reducing dimensions, while preserving the most important features of the dataset and lowering its dimensionality. The sample data collected by the selected array system composed of 12 gas sensors were subjected to PCA. First, the sample data need to be standardized:

(2) $$Z=\frac{X-\mu }{\sigma }$$

In the formula, *X* represents the original data, *μ* denotes the mean, and *σ* signifies the standard deviation.

Compute the covariance matrix for the standardized dataset *Z*:

(3) $$C=\frac{1}{n}Z^{T}Z$$

wherein *Z* denotes the standardized data matrix and *n* represents the number of samples.

The eigenvalue decomposition is then applied to the covariance matrix *C*:

(4) $$C=V⋀V^{T}$$

where *V* represents the matrix of eigenvectors, with each column being an eigenvector, and Λ represents the diagonal matrix of eigenvalues, where the diagonal elements are the eigenvalues.

The eigenvalues are sorted in descending order, and their corresponding eigenvectors define the principal components. The standardized data *Z* is then projected onto the principal component space:

(5) $$Y=ZV_{k}$$

where *V_k_* is the matrix composed of the top *k* eigenvectors, and *Y* is the dimensionality-reduced data.

**Figure 5:**
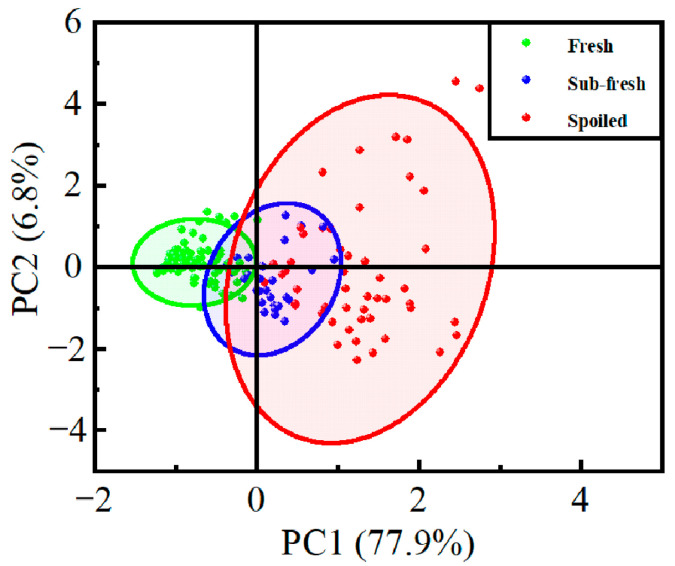
PCA of Detection Results from the Selected 12-Sensor Array System.

The analysis results of the sample data are shown in Figure 5. The contribution rate of PC1 (the first principal component) is 77.9%, while that of PC2 (the second principal component) is 6.8%. Together, the first two principal components account for 84.7% of the total variance. The notably higher variance captured by PC1 compared to PC2 indicates that the spoilage process is predominantly characterized by a single major progression axis, with PC2 representing residual non-systematic variations. This variance structure is inherent to the dataset rather than being limited by the detection capability of the sensor array, as all sensor responses were z-score standardized prior to PCA to eliminate dynamic-range dominance effects. As can be observed from the figure, a small number of data points fall outside the main sample cluster. While the distinction between fresh and spoiled samples is highly significant, the separation between sub-fresh and spoiled samples is relatively less distinct. Therefore, further analysis using multiple algorithmic models is necessary to improve the accuracy of freshness evaluation.

### 4.2. Comparison of Accuracy Under Different Classification Methods

**Figure 6:**
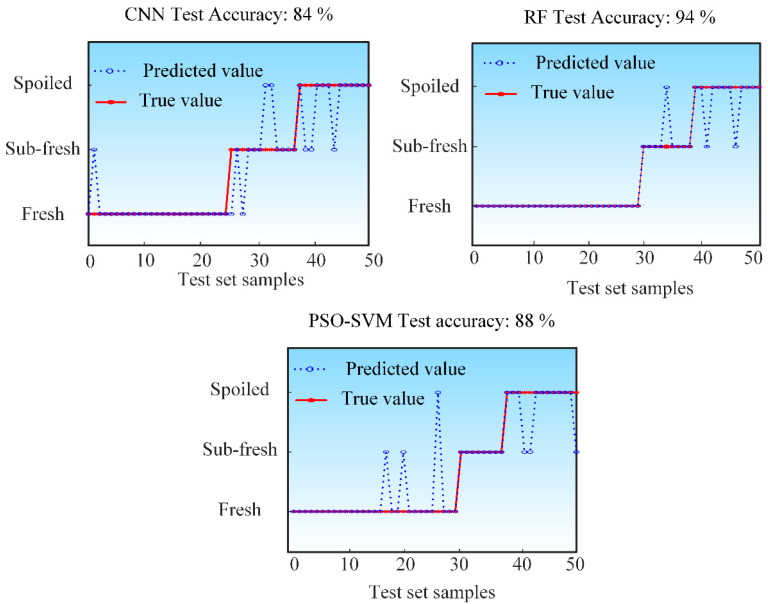
Classification accuracy of the constructed algorithmic models.

The samples collected by the gas sensor array system were randomly shuffled and divided into a training set (70% of the samples) and a test set (the remaining 30%). Three algorithmic models were developed to assess sample freshness: a Convolutional Neural Network (CNN), Random Forest (RF), and a Support Vector Machine optimized by Particle Swarm Optimization (PSO-SVM). As shown in Figure 6, the classification performance of each model is presented, with the blue line indicating predicted values and the red line representing the true values. Among them, the RF model achieved the highest classification accuracy of 94%, followed by PSO-SVM at 88%, and CNN with an accuracy of 84%.

**Figure 7:**
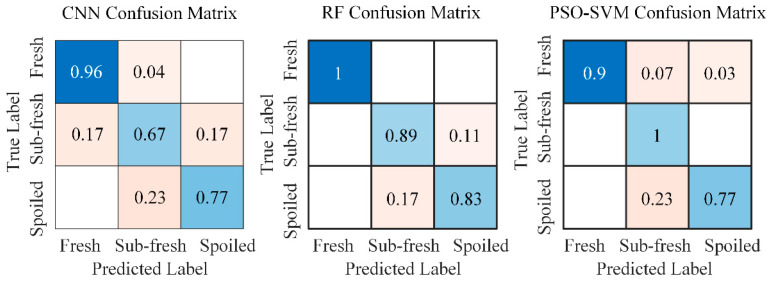
The confusion matrices of the constructed algorithmic models.

A further analysis was conducted to evaluate the per-class classification accuracy of the constructed models. Figure 7 shows the confusion matrices of the three algorithms for each category. The vertical axis represents the true labels, while the horizontal axis corresponds to the predicted labels. The diagonal entries indicate the proportion of samples where the predicted labels match the true labels, reflecting the correct classification rate. The off-diagonal elements represent misclassification rates.

As shown in Figure 7, the color gradient in the figure, from blue to pink, represents a decrease in value. Specifically, the blue color corresponds to higher values ranging from 0.5 to 1, while the pink color corresponds to lower values ranging from 0 to 0.5. The CNN model achieved a classification accuracy of 96% for fresh samples, 67% for sub-fresh samples, and 77% for spoiled samples. These results indicate that the CNN model exhibits relatively lower predictive performance for both sub-fresh and spoiled samples. The RF model attained 100% accuracy for fresh samples, 89% for sub-fresh samples, and 83% for spoiled samples. This demonstrates that the RF model performs consistently well across all freshness categories, suggesting its strong suitability for fish freshness detection. The PSO-SVM model achieved accuracies of 90% for fresh samples, 100% for sub-fresh samples, and 77% for spoiled samples. It can be observed that the PSO-SVM model shows relatively lower classification accuracy specifically for spoiled samples.

### 4.3. Optimization of the Gas-Sensor Array System

If the sensors are not properly selected, the collected data will contain significant noise and interference. This can disrupt the predefined classification rules, leading to overfitting and reducing the predictive accuracy of the algorithm. Therefore, it is necessary to optimize the sensor array to mitigate or prevent overfitting.

An analysis was conducted on the correlation between the response values of the 12 selected sensors and the degree of spoilage. The Pearson correlation coefficient is the most commonly used metric for assessing correlation. The Pearson correlation coefficient (*τ*) ranges from [−1, 1]. A value of *τ* = 1 indicates a perfect positive correlation, *τ* = −1 indicates a perfect negative correlation, and *τ* = 0 indicates no linear correlation. The formula for the Pearson correlation coefficient is as follows:

(6) $$\tau =\frac{\sum_{i=1}^{n}\left(X_{i}-\bar{X})(Y_{i}-\bar{Y}\right)}{\sqrt{\sum_{i=1}^{n}{{(X}_{i}-\bar{X})}^{2}}\sqrt{\sum_{i=1}^{n}{\left(Y_{i}-\bar{Y}\right)}^{2}}}$$

In the formula, *X* and *Y* represent the observed values of the two variables, *X_i_* and *Y_i_* denote the observed values of the *i*-th sample, and $\bar{X}$ and $\bar{Y}$ represent the means of *X* and *Y*, respectively.

To determine the significance of the correlation coefficient, a significance test is required. A two-tailed test is used to examine whether the correlation is significant. The *t* is employed to assess the significance of the Pearson correlation coefficient: a larger absolute *t* indicates that the correlation coefficient *τ* is more significant, while a smaller absolute *t* suggests that *τ* is less significant. The formula for calculating the *t* is as follows:

(7) $$t=\frac{\tau \sqrt{n-2}}{\sqrt{1-\tau^{2}}}$$

where *τ* represents the Pearson correlation coefficient and *n* denotes the sample size.

The significance level α is typically set at 0.05 or 0.01. In this chapter, the more stringent value of 0.01 is adopted. Based on the degrees of freedom *d f* = n − 2 and the significance level *α*, the critical value *t_critical_* is obtained from the *t* distribution table.

The calculated *t* value is compared with the critical value as follows:

If ∣*t*∣ > *t_critical_*, the correlation is statistically significant.

If ∣*t*∣ ≤ *t_critical_*, the correlation is not statistically significant.

**Figure 8:**
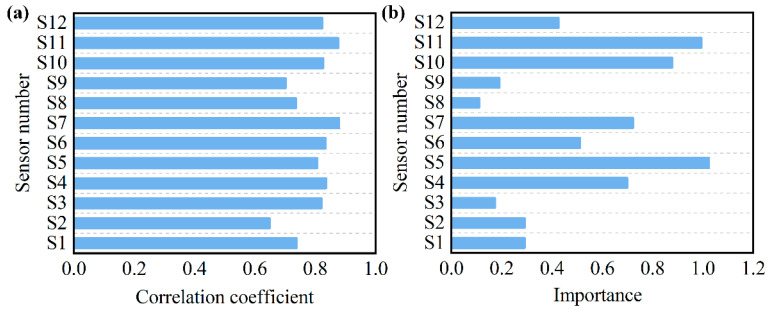
(**a**) Correlation analysis between sensor response values and sample spoilage degree; (**b**) Sensor importance analysis based on RF algorithm.

As shown in Figure 8a, the correlation analysis results indicate a highly significant relationship between the response values of each sensor and the spoilage level of the samples. Based on the correlation coefficients, sensors S7, S11, and S4 exhibited the strongest correlations, while the responses of S1, S2, S8, and S9 showed the weakest correlations with the sample spoilage level.

Figure 8b shows the importance evaluation results of the constructed random forest algorithm model for the selected 12 sensors. It can be observed that S5, S11, and S10 have the highest importance, while S1, S2, S3, S8, and S9 are relatively less important.

Based on the correlation study between sensors and sample spoilage levels, as well as the evaluation of sensor importance, it was found that sensors S1, S2, S8, and S9 exhibit relatively low levels of both correlation and importance. Therefore, these sensors can be removed to optimize the system.

**Figure 9:**
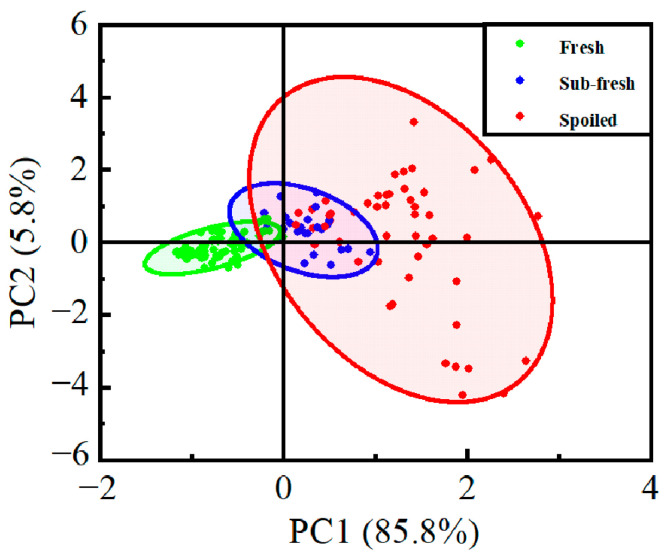
PCA of the Optimized Sensor Array System Detection Results.

PCA was performed on the detection results of the remaining eight sensors constituting the array system, as shown in Figure 9. The first principal component (PC1) accounted for 85.8% of the variance, while the second principal component (PC2) explained 5.8%. Together, the first two principal components captured 91.6% of the total variance, representing a 6.9% improvement compared to the PCA results from the original 12-sensor array system. The further increased dominance of PC1 after sensor pruning reinforces that the data variance is primarily unidirectional along the spoilage trajectory, with the low PC2 contribution reflecting the fundamental data structure rather than sensor detection limits. Under our measurement protocol featuring headspace equilibration and stable response acquisition, potential LOD effects at early spoilage stages are minimized. Most data points are contained within the sample cluster, with a few outliers located relatively close to the main distribution. The distinction between freshness levels has been further enhanced compared to previous analyses. The PCA results effectively differentiate between fresh and spoiled samples, as well as between fresh and moderately fresh samples. However, the separation between moderately fresh and spoiled samples remains insufficiently distinct, necessitating further development of algorithmic models to improve recognition accuracy.

**Figure 10:**
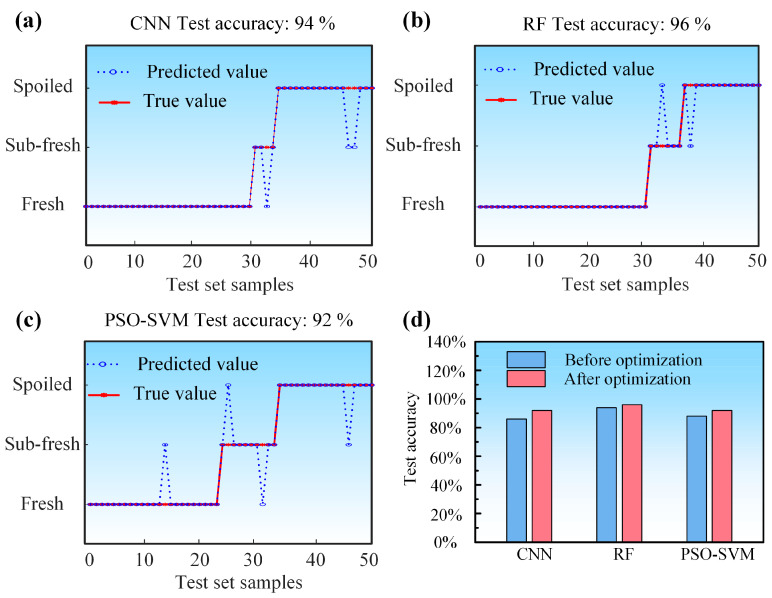
Classification accuracy of the algorithm model constructed from sample data of the optimized sensor array system: (**a**) CNN, (**b**) RF, (**c**) PSO-SVM; (**d**) Comparison of prediction accuracy between algorithm models before and after optimization.

The sample data from the optimized sensor array system was analyzed using CNN, RF, and PSO-SVM algorithms, with all model parameters maintained consistent with those used before optimization. As shown in Figure 10, among the models trained on the optimized data, the RF model achieved the highest prediction accuracy of 96%. The CNN attained an accuracy of 94%, while the accuracy of the PSO-SVM model improved to 92%. Compared to the performance with the pre-optimization sensor array system, all models exhibited higher prediction accuracy after optimization. These results demonstrate that the optimization of the sensor array system effectively reduced redundant information and enhanced the overall recognition capability of the system.

**Figure 11:**
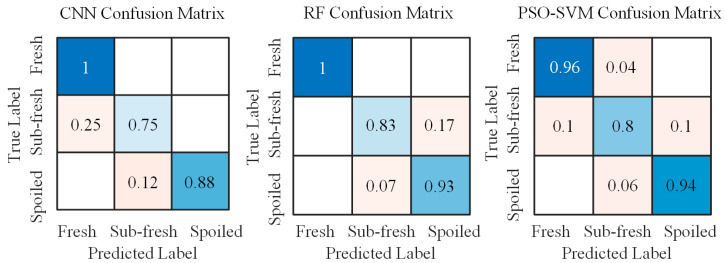
The confusion matrices of the algorithm models constructed after optimization.

Figure 11 presents the confusion matrices of the algorithm models built with the optimized sensor array system. The RF model attains 100% accuracy for fresh, 83% for sub-fresh, and 93% for spoiled, with most residual errors occurring precisely at the sub-fresh vs. spoiled boundary. The main reasons for this are analyzed below.

Fish spoilage is a gradual biochemical process rather than a step change. Key markers (e.g., trimethylamine, ammonia, hydrogen sulfide, alcohols) co-exist with shifting concentrations, producing partially overlapping chemical profiles and, in turn, similar sensor responses near the transition zone.

Even within a single species, differences in size, fat content, initial microbial load, and other attributes introduce sample-to-sample variability that is most pronounced around the spoilage threshold, further blurring class boundaries.

In summary, these findings indicate that fresh samples can be identified reliably, whereas the sub-fresh vs. spoiled boundary remains the most challenging due to the continuity of spoilage chemistry and heightened biological variability near the threshold. In the present work, we therefore report per-class metrics and confusion matrices (Figure 11) to make this limitation explicit and to guide practical deployment (e.g., threshold setting). Looking ahead, we will augment the gas-sensor array with near-infrared spectroscopy (molecular-vibrational information) and computer vision (macro-morphological cues) to construct a multimodal dataset; because these modalities are complementary to volatile-profile sensing, their fusion via machine-learning methods is expected to sharpen the decision boundary at the transition zone, yield more reliable freshness indicators, and improve robustness to sample-to-sample variability.

### 4.4. Practicality and Cost Trade-Offs

For portable or appliance-grade use, the dominant complexity/cost drivers are the number of heated sensing channels, thermal drivers and control, gas-handling hardware (pumps/valves/manifolds), and compute/IO (ADC/MCU/multiplexers). Consistent with our selection analysis, feature-guided channel reduction can lower bill-of-materials, power consumption, physical footprint, and calibration burden while preserving discriminative capability. In practice, duty-cycled operation and lightweight on-device inference, multiplexed readout, and streamlined gas paths (e.g., passive diffusion or a single micro-blower) further enable compact, low-power configurations suitable for real-world deployment.

## 5. Conclusions

This work addresses the selectivity limitations of oxide-semiconductor gas sensors in mixed-gas environments by exploiting complementary cross-sensitivity within a reconfigurable multi-channel array. The platform supports a configurable sensor panel with flexible electrode options, independent thermal management, and programmable gas handling, enabling adaptation to diverse deployment scenarios. The study substantiates that coupling such arrays with feature extraction and supervised-learning strategies can provide reliable freshness assessment under realistic headspace conditions without relying on single-sensor specificity. Moreover, feature-guided channel selection offers a principled route to reducing hardware complexity and mitigating overfitting while preserving discriminative power. Overall, this work provides a methodological and system-level foundation for applying gas-sensor arrays to complex mixture discrimination and practical food-quality monitoring, and points to multimodal fusion and domain-aware modeling as promising directions to further resolve boundary cases.

## Figures and Tables

**Table 1:** Gas sensor array and its properties.

Sensor Number	Brand	Model	Target Gas	Heating Voltage (V)	Detection Range (ppm)	Number of Electrodes
S1	Figaro	TGS2600	Hydrogen, Alcohol	5	1~30	4
S2	Figaro	TGS2611	Methane, Natural gas	5	500~10,000	4
S3	Figaro	TGS2602	Ammonia, Hydrogen sulfide	5	1~30	4
S4	Figaro	TGS2603	Trimethylamine, Methyl mercaptan	5	1~10	4
S5	Figaro	TGS2620	Ethanol, Organic solvents	5	50~5000	4
S6	Winsen	WSP2110	Toluene, Formaldehyde	5	1~50	4
S7	Winsen	WSP7110	Hydrogen sulfide	5	0~50	4
S8	Winsen	MP702	Ammonia	5	0~100	4
S9	Self-developed	In_2_O_3_ nanocuboids	Triethylamine	4	0.5~100	6
S10	Self-developed	bayberry-like In_2_O_3_	Trimethylamine	3.5	0.5~100	6
S11	Self-developed	flower-like In_2_O_3_	Trimethylamine	3	0.3~100	6
S12	Figaro	TGS 832	Halogenated hydrocarbons, VOCs	5	1~50	6

**Table 2:** Classification of Fish Freshness Status.

TVB-N Content (mg/kg)	Freshness
≤50	Fresh
50~250	Sub-fresh
≥250	Spoiled

## Data Availability

The data used in the analysis presented in the paper will be made available, subject to the approval of the data owner.

## Supplementary Materials

The following supporting information can be downloaded at: https://www.mdpi.com/article/10.3390/s25196212/s1, Sensor selection criteria; Table S1: Comparison of fish freshness detection techniques.

## Abbreviations

The following abbreviations are used in this manuscript:

VOCs	Volatile Organic Compounds
TVB-N	Total Volatile Basic Nitrogen
PCA	Principal Component Analysis
CNN	Convolutional Neural Network
RF	Random Forest
PSO-SVM	Support Vector Machine optimized by Particle Swarm Optimization
