# Supplementary material for: Reconfigurable Multi-Channel Gas-Sensor Array for Complex Gas Mixture Identification and Fish Freshness Classification
Source: Sensors (Basel). 2025 Oct 7;25(19):6212. doi: 10.3390/s25196212 (PMC12526641; doi:10.3390/s25196212)
Supplement: Supplementary file 1 [file sensors-25-06212-s001.zip › sensors-3906602-supplementary.pdf]

Supporting Information

Reconfigurable Multi-Channel Gas-Sensor Array for Complex Gas Mixture Identification and Fish Freshness Classification

He Wang [1,2], Dechao Wang [1,2], Hang Zhu [1,2,*] and Tianye Yang [1,2,*]

[1] National Key Laboratory of Automotive Chassis Integration and Bionics, School of Mechanical and Aerospace Engineering, Jilin University, Changchun, 130022, People's Republic of China.

[2] Key Laboratory of CNC Equipment Reliability, Ministry of Education, Jilin University, Changchun 130022, China

* Correspondence: hangzhu@jlu.edu.cn; yangty@jlu.edu.cn

1. Sensor selection criteria

(1) Application-driven coverage of expected volatiles

Headspace during fish spoilage is dominated by amines (TMA/TEA) and ammonia, with contributions from sulfur species, alcohols, aldehydes, and some alkanes. We therefore mapped each sensor to a defined “role” to ensure the array’s dynamic range and operating windows overlap these targets:

- Hydrogen sulfide: TGS2602 and WSP7110
- Ethanol: TGS2600 and TGS2620
- Ammonia: TGS2602 and MP702
- Trimethylamine: TGS2603, bayberry-like In_2O_3, flower-like In_2O_3
- Triethylamine: In_2O_3 nanocuboids
- Aldehydes: WSP2110
- Alkanes: TGS2611
- Broad VOCs: TGS832, which exhibit good responses to various VOC gases

(2) Objective, multi-criteria screening

From a broader candidate list, we retained devices that satisfied practical constraints for this use case: (i) LOD and linear range aligned with reported headspace levels for spoilage markers; (ii) response/recovery times compatible with our measurement cycle; (iii) humidity tolerance, given the intrinsically high RH of fish samples; (iv) operating temperature and power suited to compact integration; (v) stability, packaging, and supply continuity for reproducibility. Candidates failing these criteria (e.g., excessive temperature, insufficient range, or poor humidity robustness) were excluded.

(3) Commercial vs. self-developed: complementary roles rather than redundancy

Commercial sensors (TGS/WSP/MP series) provide reliable baseline coverage across alcohols/aldehydes/alkanes and ammonia with proven manufacturability. Our self-developed In_2O_3 variants were retained to strengthen low-ppm amine sensitivity (TMA/TEA) and to mitigate high-humidity interference typical of fish matrices—two gaps that were difficult to close with commercial-only panels. The combined panel therefore improves coverage and robustness without requiring single-analyte exclusivity.

(4) Cross-sensitivity leveraged by design

Rather than demanding absolute specificity, we intentionally use partially overlapping response patterns so that the array forms a robust "odor fingerprint" interpretable by the pattern-recognition framework already presented. This strategy enhances resilience to sensor drift and environmental variability.

In summary, the sensor panel was assembled through a transparent, application-driven screening process. We prioritized coverage of spoilage volatiles—amines (TMA/TEA) and NH_3, sulfur species, alcohols, aldehydes, and alkanes—and combined commercial devices with self-developed In_2O_3 variants to enhance low-ppm amine sensitivity and humidity robustness. By deliberately leveraging cross-sensitivity and complementary operating windows, the array yields an information-rich odor fingerprint. This design enables reliable freshness assessment via pattern-recognition methods rather than relying on single-sensor specificity.

2. Comparison of fish freshness detection techniques

For fish freshness assessment, common approaches include sensory evaluation, physical-property analyses, chemical assays, and microbiological tests. Table S1 provides a detailed comparison of the principal techniques currently in use. As the comparison indicates, each method has its own strengths in operational convenience, accuracy, detection efficiency, and cost; however, gas-sensor-based methods offer the most balanced overall performance. In particular, they combine high detection accuracy and real-time capability with user-friendly operation and moderate cost, making them highly competitive for practical applications.

Table S1. Comparison of fish freshness detection techniques.

Detection method	Operational complexity	Cost	Scope of application	Detection efficiency	Accuracy	Technology maturity
Sensory evaluation	Simple	Low	Broad	High	Low	Medium
Physical property analysis	Moderate	Medium	Moderate	Medium	Medium	Medium
Chemical analysis	Complex	Medium	Moderate	Medium	High	High
Microbiological testing	Complex	High	Limited	Low	High	High
Gas sensor detection	Moderate	Medium	Broad	High	High	Low
